# Heteroaryl Chalcones: Design, Synthesis, X-ray Crystal Structures and Biological Evaluation

**DOI:** 10.3390/molecules181012707

**Published:** 2013-10-15

**Authors:** C. S. Chidan Kumar, Wan-Sin Loh, Chin Wei Ooi, Ching Kheng Quah, Hoong-Kun Fun

**Affiliations:** 1 X-ray Crystallography Unit, School of Physics, Universiti Sains Malaysia, Penang 11800 USM, Malaysia; E-Mails: chidankumar@gmail.com (C.S.C.K.); wansin_loh@live.com (W.-S.L.); ooichinwei88@gmail.com (C.W.O.); ckquah@usm.my (C.K.Q.); 2 Department of Chemistry, Alva’s Institute of Engineering & Technology, Mijar, Moodbidri 574225, India; 3 Department of Pharmaceutical Chemistry, College of Pharmacy, King Saud University, Riyadh 11451, Saudi Arabia

**Keywords:** heteroaryl, antimicrobial, reducing, substituent, overlay

## Abstract

Chalcone derivatives have attracted increasing attention due to their numerous pharmacological activities. Changes in their structures have displayed high degree of diversity that has proven to result in a broad spectrum of biological activities. The present study highlights the synthesis of some halogen substituted chalcones **3**(**a**–**i**) containing the 5-chlorothiophene moiety, their X-ray crystal structures and the evaluation of possible biological activities such as antibacterial, antifungal and reducing power abilities. The results indicate the tested compounds show a varied range of inhibition values against all the tested microbial strains. Compound **3c** with a *p*-fluoro substituent on the phenyl ring exhibits elevated antimicrobial activity, whereas the compounds **3e** and **3f** displayed the least antimicrobial activities. The compounds **3d**, **3e**, **3f** and **3i** showed good ferric and cupric reducing abilities, and the compounds **3b** and **3c** showed the weakest reducing power in the series.

## 1. Introduction

Chalcones are important constituents of many natural products. They are abundant in edible plants where they are considered to be the precursors of flavonoids and isoflavonoids. There is growing interest in the pharmacological potential of chalcones which constitute an important group of natural and synthetic products that have been screened for a wide range of pharmacological activities such as antibacterial [1,2], antitumor [3,4,5], anti-inflammatory [6,7,8,9], antifungal [10] and antioxidant properties [11,12,13,14,15]. Chalcones are also well known intermediates for synthesizing various heterocyclic compounds. Several methods have been reported for the synthesis of chalcones, among them aldol condensation and Claisen-Schmidt condensation between aryl ketones and aromatic aldehydes in acidic or basic media still occupy prominent positions. Chalcones are characterized by possessing an enone moiety between two aromatic rings. Elemental sulfur has been well known to act as an antifungal agent for a long time. Several naturally occurring antifungal agents are also known to contain sulfur [16]. Many researchers have reported chalcones containing sulfur either as a part of heteroaryl ring (thiophene) or as a side chain (thiomethyl group). Tomar *et al.* have reported the synthesis and antimicrobial activity of chalcones containing the 2,5-dichlorothiophene moiety [17]. Seema *et al.* have reported the synthesis and biological evaluation of α,β-unsaturated ketones as potential antifungal agents [18]. Tran *et al.* have reported the synthesis and antibacterial activity of some heterocyclic chalcone analogues alone and in combination with antibiotics [19]. Ranganathan *et al.* have reported the synthesis and antimicrobial studies of some of the 5-chloro-2-acetylthiophene chalcones [20]. In continuation of our research work on the X-ray crystal structure studies of 5-chlorothiophene chalcone analogues [21], in which an effort has been made to diversify the pharmacological activities by exploring the structural diversity of conventional chalcones, a series of halogen(s)-substituted chalcone analogues containing electron-rich thiophene heterocycles were synthesized. This was done with the expectation that the derivatives with halogen functionalities at different positions on the phenyl ring would be even more potent as antimicrobial agents than the starting compounds. Herein we report the synthesis of some novel halogen substituted chalcone analogues with 5-chlorothiophene moiety using the conventional base-catalyzed Claisen-Schmidt condensation, their crystal structures and the evaluation of their antimicrobial, ferric ion and cupric ion reducing power abilities.

## 2. Results and Discussion

**Scheme 1:**
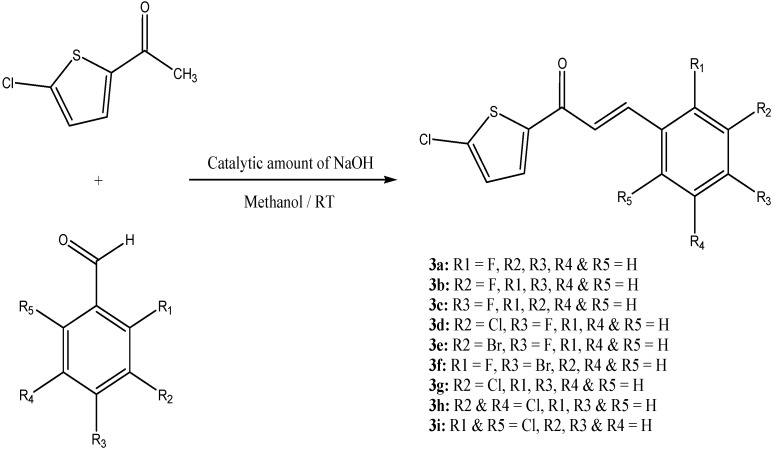
Synthesis of heterocyclic chalcone analogues.

Chalcones, a versatile class of natural and synthetic compounds, have attracted the interest of researchers for their wide range of biological activities. Chalcones containing the thiophene moiety are supposed to further enhance these activities. The best known method for the synthesis of chalcones is the Claisen-Schmidt condensation between acetophenone and benzaldehyde in basic media. New chalcones have been designed and synthesized by the reaction of 2-acetyl-5-chlorothiophene with halogen-substituted benzaldehydes using a catalytic amount of NaOH in methanol as shown in Scheme 1.

### 2.1. X-Ray Crystal Structure Description for Compounds 3a–3i

**Figure 1:**
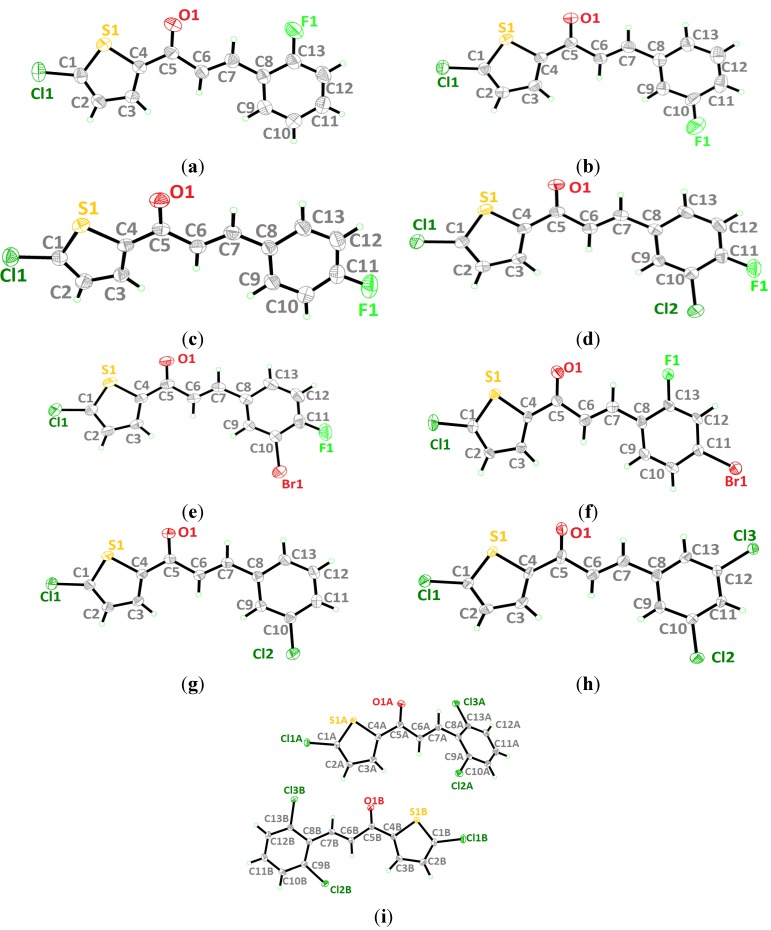
(**a**)–(**i**). Ortep diagram of compounds **3a** to **3i** (**3h** and **3i** are drawn at 50% ellipsoids for non-hydrogen atoms and the remaining compounds are drawn at 30% ellipsoids for non-hydrogen atoms).

**Figure 2:**
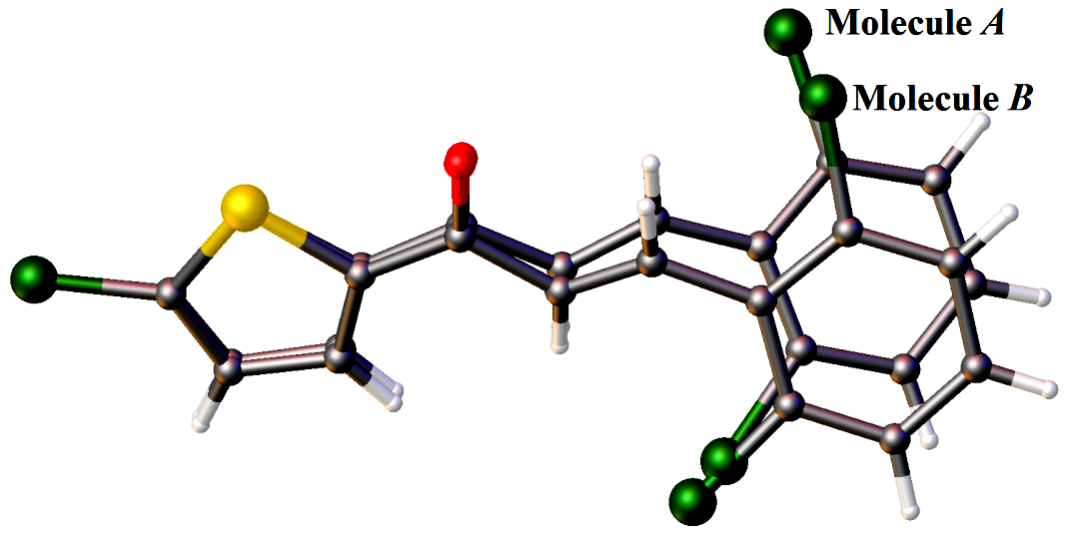
Overlay of molecules *A* and *B* in compound **3i**, calculated using the chlorothiophene moiety.

The crystal structures of **3**(**a**–**i**) are as shown in Figure 1. Table 1 lists the crystallographic data for all the nine compounds. Four out of nine compounds were crystallized in orthorhombic system, where **3a** in space group *Pca*2_1_, **3c**, **3d** and **3e** in space group *Pbca*. The remaining compounds (**3b**, **3f**, **3g**, **3h** and **3i**) crystallized in monoclinic system with space group *P*2_1_*/c*. The overall conformation of the molecules (**3a**–**i**) can be described by the dihedral angles formed between the thiophene (S1/C1—C4) and the benzene (C8—C13) rings. The respective parameters are listed in Table 2. As seen from the table, the conformation of the molecules is almost planar except for compound **3i** and it can be related to the presence of substituent in the -*ortho* position of the benzene ring. Compound **3i** contains two crystallographically independent molecules (molecules *A* & *B*). The thiophene and benzene rings in compounds **3i** are relatively far from planarity as the values of dihedral angles between them are 45.68 (11)° in molecule *A* and 24.00 (11)° in molecule *B*. The overlay of both molecules over all atoms is shown in Figure 2, with the r.m.s value of 0.339 Å.

**Figure 3:**
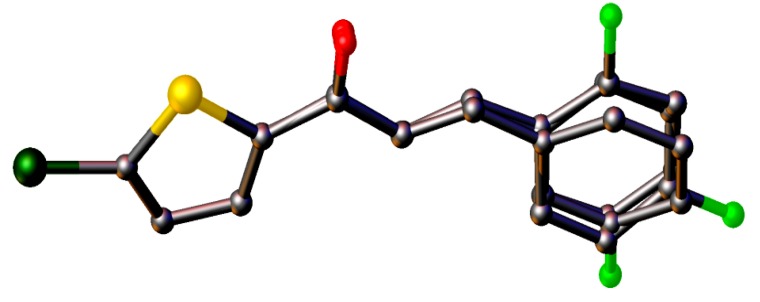
Overlay of all non-H atoms in compounds **3a**, **3b** and **3c**, calculated using the chlorothiophene moiety.

**Figure 4:**
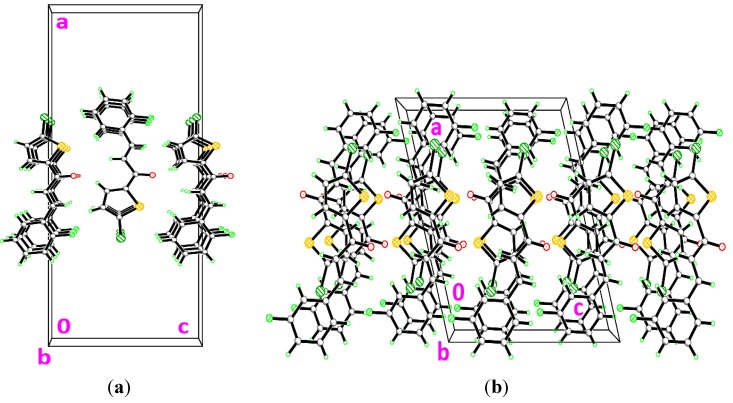
Crystal structures of (**a**) **3a** and (**b**) **3b**. The molecules are stacked along the *b*-axis.

**Figure 5:**
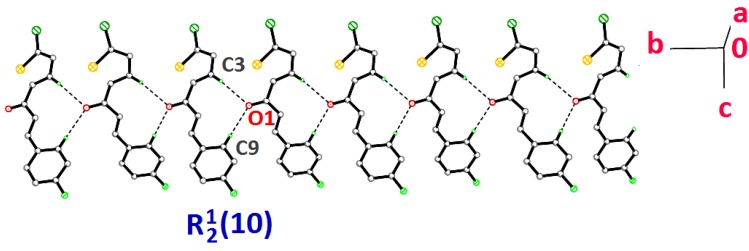
Crystal structure of **3c** forming chains along the *b*-axis. Dashed lines indicate the intermolecular hydrogen bonds.

Compounds **3a**, **3b** and **3c** differ structurally from each other by having the fluoro-substituent at -*ortho*, -*meta* and -*para* positions of the benzene rings, respectively. The planarity of the molecules are indicated by the dihedral angles as shown in Table 2, ranging from 5.5 (2) to 15.1 (3)°. Figure 3 shows the overlaid molecules over all the non-H atoms, calculated using the chlorothiophene moiety with the r.m.s values of 0.103 Å for **3a**/**3b** and **3a**/**3c**, and 0.051 Å for **3b**/**3c**. There is no classical hydrogen bond found in compounds **3a** and **3b**. The formation of short intra-molecular S···O contacts of 2.926(3) and 2.933(4) Å which is 0.39 Å shorter than the sum of van der Waals radii of the sulfur and oxygen atoms, in both **3a** and **3b**, could be due to the charge delocalization into the carbonyl group from the thiophene ring. This helps to stabilize the molecular structure. In the crystal structure (Figure 4a), molecules in **3a** are stacked along the *b*-axis, with alternative stacks forming an inverted head-to-tail arrangement. In **3b**, the molecules stacked on each other in an inverse fashion along the *b*-axis (Figure 4b), whereas in compound **3c**, the molecules are linked into chains along *b*-axis as illustrated in Figure 5, *via* intermolecular C3—H3A···O1 and C9—H9A···O1 hydrogen bonds (Table 3), forming 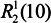 ring motifs [22].

**Table 1:** Crystal data and parameters for structure refinement of **3a** to **3i**.

Compound	3a	3b	3c	3d	3e	3f	3g	3h	3i
CCDC deposition number	939875	939876	939877	942741	942742	948856	944072	946445	948855
Molecular formula	C_13_H_8_ClFOS	C_13_H_8_ClFOS	C_13_H_8_ClFOS	C_13_H_7_Cl_2_FOS	C_14_H_7_BrClFOS	C_13_H_7_BrClFOS	C_13_H_8_Cl_2_OS	C_13_H_7_Cl_3_OS	C_13_H_7_Cl_3_OS
Molecular weight	266.70	266.70	266.70	301.15	345.61	345.61	283.15	317.60	317.60
Crystal system	Orthorhombic	Monoclinic	Orthorhombic	Orthorhombic	Orthorhombic	Monoclinic	Monoclinic	Monoclinic	Monoclinic
Space group	*Pca*2_1_	*P*21/*c*	*Pbca*	*Pbca*	*Pbca*	*P*21/*c*	*P*21/*c*	*P*21/*c*	*P*21/*c*
*a* (Å)	27.963 (9)	14.282 (6)	7.9809 (8)	7.4870 (9)	7.476 (2)	3.8704 (7)	14.5437 (10)	14.9864(10)	30.3418 (16)
*b* (Å)	3.9233 (15)	7.698 (4)	11.0207 (11)	11.6186 (14)	11.724 (3)	31.953 (6)	7.5227 (6)	3.8085 (3)	3.7985 (2)
*c* (Å)	11.146 (4)	11.376 (5)	27.518 (3)	29.239 (4)	30.009 (8)	10.273 (2)	11.7604 (9)	24.6297 (13)	23.4311 (12)
α (°)	90	90	90	90	90	90	90	90	90
β (°)	90	104.430	90	90	90	95.243 (3)	104.721 (1)	115.264 (3)	109.297 (1)
γ (°)	90	90	90	90	90	90	90	90	90
*V (*Å^3^)	1222.8 (7)	1211.3 (9)	2420.3 (4)	2543.4 (5)	2630.4 (13)	1265.2 (4)	1244.44 (16)	1271.30 (15)	2548.8 (2)
*Z*	4	4	8	8	8	4	4	4	8
*D*_calc_ (g cm^−3^)	1.449	1.462	1.464	1.573	1.745	1.814	1.511	1.659	1.655
Crystal dimensions (mm)	0.93 × 0.18 × 0.05	0.25 × 0.25 × 0.05	0.79 × 0.37 × 0.04	0.75 × 0.52 × 0.10	0.58 × 0.28 × 0.09	0.89 × 0.12 × 0.09	0.50 × 0.23 × 0.08	0.51 × 0.10 × 0.05	0.60 × 0.10 × 0.10
Colour	Colourless	Colourless	Colourless	Colourless	Colourless	Colourless	Colourless	Colourless	Colourless
μ(mm^−1^)	0.47	0.48	0.48	0.67	3.48	3.62	0.67	0.87	0.86
Radiation λ (Å)	0.71073	0.71073	0.71073	0.71073	0.71073	0.71073	0.71073	0.71073	0.71073
*T*_min_/*T*_max_	0.668/0.975	0.889/0.975	0.704/0.980	0.633/0.935	0.237/0.752	0.141/0.744	0.733/0.946	0.665/0.955	0.624/0.922
Reflections measured	8618	9071	24679	16026	16116	2855	11650	11375	20108
Ranges/indices (*h*, *k*, *l*)	−37, 39; −5, 5; −15, 15	−16, 16; −9, 9; −13, 13	−11, 11; −15, 15; −38, 37	−10, 10; −14, 16; −41, 37	−10, 9; −12, 16; −42, 31	−5, 5; −41, 41; −1, 13	−18, 18; −9, 9; −15, 15	−19, 19; −4, 4; −31, 31	−39, 39; −4, 4; −30, 30
θ limit (°)	2.3–22.4	3.0–19.5	3.2–25.3	2.8–26.4	2.7–25.3	2.8–29.8	2.9–29.0	2.8–30.1	2.9–29.9
Unique reflections	3368	2126	3540	3738	3816	2855	2829	2894	5762
Observed reflections (*I* > 2σ(*I*))	1899	1133	2016	2486	1935	2624	2312	2607	4784
Parameters	154	154	154	163	163	164	154	163	325
Goodness of fit on *F*^2^	0.99	1.00	1.03	1.03	1.01	1.09	1.04	1.05	1.03
*R*_1_, *wR*_2_ [*I* ≥ 2σ(*I*)]	0.049, 0.136	0.059, 0.191	0.047, 0.135	0.041, 0.135	0.044, 0.136	0.051, 0.127	0.030, 0.101	0.046, 0.120	0.033, 0.091

**Table 2:** The dihedral angles formed between the chlorothiophene and benzene rings.

Compound	Dihedral angle between two rings (°)
**3a**	5.5 (2)
**3b**	15.1 (3)
**3c**	14.98 (13)
**3d**	9.45 (9)
**3e**	6.58 (16)
**3f**	1.2 (2)
**3g**	16.40 (8)
**3h**	2.07 (15)
**3i**	*Molecule* *A*-45.68 (11) *Molecule B*-24.00 (11)

**Table 3:** Hydrogen bond geometries for the compounds **3c**, **3f**, **3h** and **3i**.

*D*–H···*A*	*d*(*D*–H) (Å)	*d*(H···*A*)(Å)	*d*(*D*···*A*)(Å)	*Angle*(*D*–H···*A*)(°)
**3c**				
C3—H3A···O1 ^i^	0.93	2.56	3.438(3)	157
C9—H9A···O1 ^i^	0.93	2.54	3.474(3)	179
**3f**				
C2—H2A···F1 ^ii^	0.95	2.40	3.295 (5)	156
C3—H3A···O1 ^ii^	0.95	2.48	3.381 (5)	158
**3h**				
C7—H7A···O1 ^iii^	0.93	2.49	3.337(4)	151
C13—H13A···O1 ^iii^	0.93	2.48	3.300(4)	148
**3i**				
C2B—H2BA···O1A ^iv^	0.95	2.50	3.209(3)	131

Symmetry code: (i) −*x*+1/2, *y*−1/2, *z*; (ii) *x*−1, −*y*+1/2, *z*−1/2; (iii) −*x*+1, −*y*+2, −*z*; (iv) *x*, −*y*+5/2, *z*+1/2.

**Figure 6:**
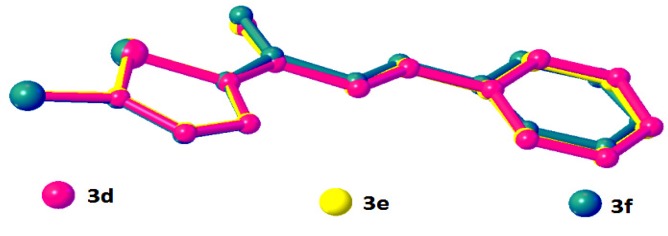
Overlay of compounds **3d**, **3e** and **3f** excluding halogen substituents at benzene rings and H-atoms.

**Figure 7:**
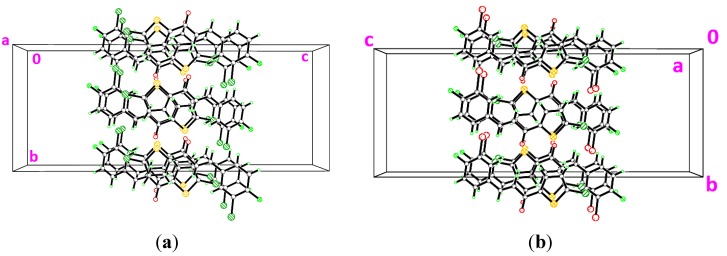
Crystal structures of (**a**) **3d** and (**b**) **3e**, showing the stacking along the *a*-axis.

**Figure 8:**
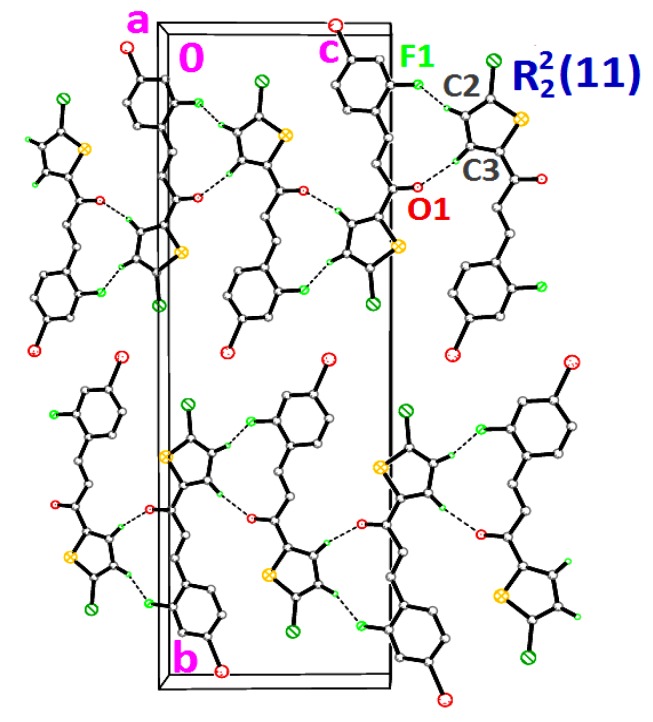
Crystal structure of **3f**, showing the chains along the *c*-axis. Dashed lines indicate the intermolecular hydrogen bonds.

Compounds **3d**, **3e** and **3f** differ from **3a**–**3c** by the additional halogen substituent at the benzene rings. The chlorothiophene and the benzene rings of **3d**–**3f** are approximately planar, with dihedral angles ranging from 1.2 (2) to 9.45 (9)°. The conformations of these three molecules are very analogous as indicated by the overlay, excluding H atoms and the halogen substituents on the benzene rings (Figure 6) with r.m.s values of 0.033 Å for **3d**/**3e**, 0.111 Å for **3d**/**3e** and 0.088 Å for **3e**/**3f**. Compounds **3d** and **3e** were observed to possess no significant hydrogen bonds. They are stabilized by the formation of short intramolecular S···O contacts of 2.933 (4) and 2.929 (3) Å, respectively, which is 0.39 Å shorter than the sum of van der Waals radii of the sulfur and oxygen atoms. In the crystal structure, molecules in **3d** and **3e** stacked along the *a*-axis, as depicted in Figure 7a,b, respectively. In the crystal packing of **3f** (Figure 8), intermolecular C2—H2A···F1 and C3—H3A···O1 hydrogen bonds (Table 3) link the molecules into chains along the *c*-axis, generating 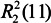 ring motifs [22].

**Figure 9:**
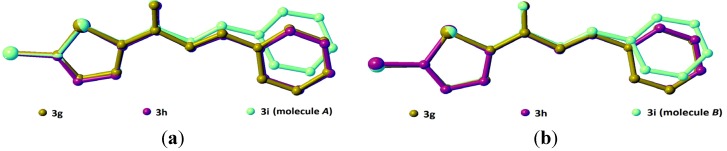
Overlay of compounds **3g**, **3h** and (**a**) **3i** with only molecule *A* and (**b**) **3i** with only molecule *B* where their chloro-substituents were excluded.

**Figure 10:**
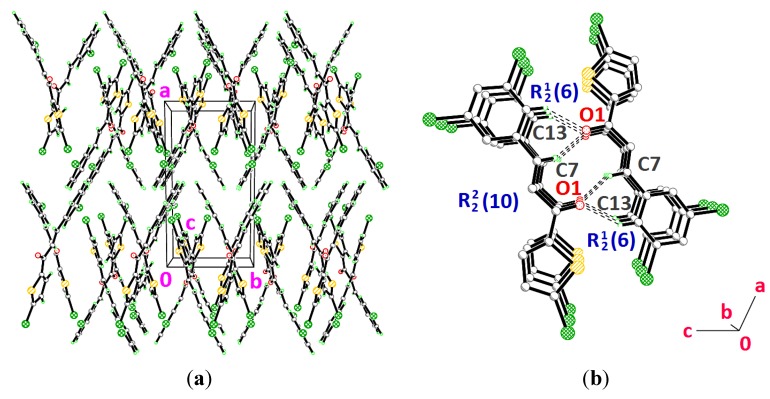
Crystal structures of (**a**) **3g** viewed along the *c*-axis (**b**) **3h** viewed along the *b*-axis. Dashed lines indicate the intermolecular hydrogen bonds.

**Figure 11:**
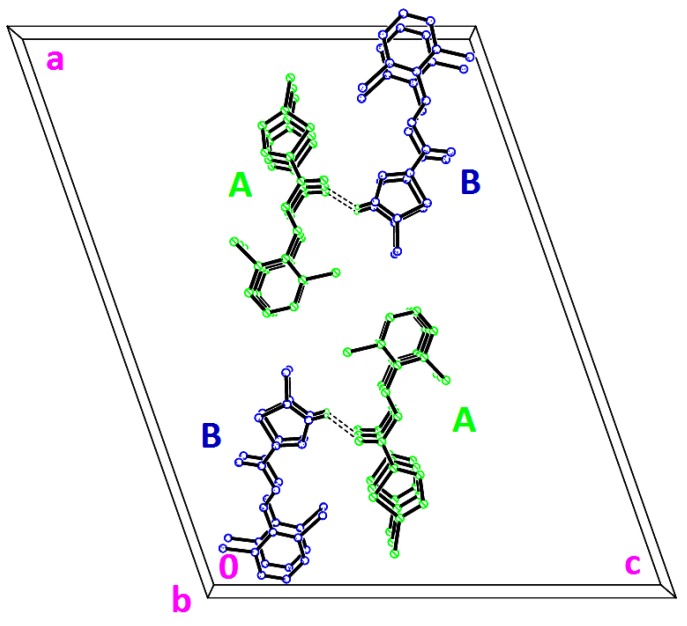
Crystal packing of **3i**, viewed along the *b*-axis. Dashed lines indicate the intermolecular hydrogen bonds.

Compounds **3g**, **3h** and **3i** present one or two chloro-substituents at the benzene rings. Compounds **3g** and **3h** are approximately planar as indicated in Table 2. Figure 9a,b show the overlays of all non-H atoms of **3g**/**3h**, **3g**/**3i** and **3h**/**3i**, calculated using the chlorothiophene moiety excluding their chloro-substituents. The r.m.s values given by the overlays of **3g**/**3h**, **3g**/**3i** (molecule *A*) and **3h**/**3i** (molecule *A*) are 0.167, 0.323 and 0.447 Å, respectively. These values are compared with another overlay (Figure 9b), taking only molecule *B* into the calculation and the r.m.s values given by the overlays of **3g**/**3i** (molecule *B*) and **3h**/**3i** (molecule *B*) are 0.205 and 0.248 Å, respectively. In general, due to intermolecular hydrogen bonding of molecules *A* and *B* in compound **3i**, they are very much deviated from the molecules of compounds **3g** and **3h**. Crystal packing diagram of **3g** with no significant hydrogen bonds is depicted in Figure 10a. However, the molecules in **3g** are stabilized by the short intra-molecular S···O contacts of 2.9348(14) Å, which is 0.39 Å shorter than the sum of van der Waals radii of the sulfur and oxygen atoms. The molecules in compound **3h** (Figure 10b) are linked to form dimers with 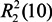 ring motifs [22] *via* intermolecular C7—H7A···O1 hydrogen bonds (Table 3). These sets of dimers are further connected by intermolecular C13—H13A···O1 hydrogen bonds (Table 3) into another two 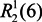 ring motifs. The molecules stacked along the *b*-axis. In the crystal structure of **3i** (Figure 11), molecules *A* highlighted in green are joined with the adjacent molecules *B* highlighted in blue through intermolecular C2B—H2BA···O1A hydrogen bonds (Table 3).

### 2.2. *In Vitro* Antimicrobial Activity

**Figure 12:**
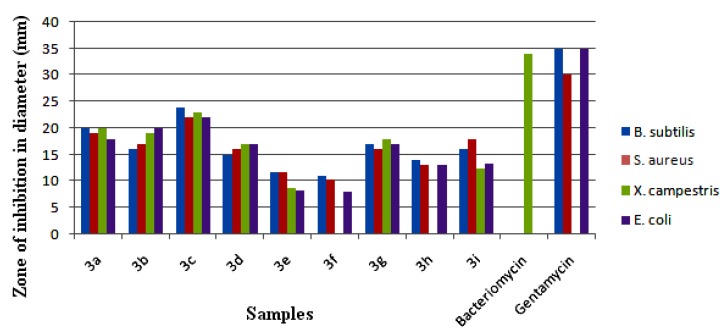
*In vitro* antibacterial activities of **3**(**a**–**i**).

**Figure 13:**
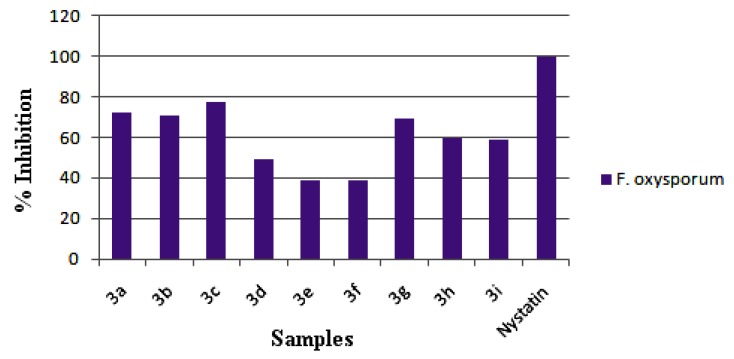
*In vitro* antifungal activity of **3**(**a–i**).

The synthesized compounds **3**(**a**–**i**) were evaluated for their *in vitro* antibacterial activity and the results are compared with bacteriomycin and gentamycin used as standard drugs (Figure 12). All the tested compounds displayed varied antibacterial activity against four pathogenic bacterial strains: *Bacillus subtilis* MTCC 121, *Staphylococcus aureus* MTCC, *Xanthomonas campestris* MTCC 7908 and *Escherichia coli* MTCC 7410. Among the series **3**(**a**–**i**), compound **3c** showed an elevated antibacterial activity against the tested bacterial strains and the compounds **3a**, **3b** and **3g** showed good antibacterial activity against all the tested organisms. The compounds **3d**, **3h** and **3i** displayed moderate activity, while the compounds **3e** and **3f** showed the least inhibitory activity in the series. Further, all the synthesized compounds **3**(**a**–**i**) were evaluated for their *in vitro* antifungal activity against *Fusarium oxysporum*. The results are shown and compared with the standard drug nystatin in Figure 13. Compounds **3a**, **3b** and **3c** showed good antifungal activity against *F. oxysporum* when compared to other compounds in the series. Compounds **3d**, **3g**, **3h** and **3i** were found to be moderately active against the tested fungal strain. The compounds **3e** and **3f** were less active.

A close survey of the antimicrobial results indicates the compounds under test show a varied range of inhibition values against all the tested bacterial and fungal strains. The elevated activity of the compound **3c** may be due to the *p*-fluoro substituent on the phenyl ring. Moreover the good activity of compounds **3a**, **3b** and **3g** can be attributed to the *o*-fluoro, *m*-fluoro and *m*-chloro substitution, respectively. The least activity of compounds **3e** and **3f** against the tested fungal strain may be due to the presence of bromo substituents. An increase in the number of halogen substituents on the phenyl ring results in a decrease in the antimicrobial activity of the tested compounds. The activities of the reported compounds against the tested bacterial strains were in the order **3c** > **3a** > **3b** > **3g** > **3h** > **3i** > **3d** > **3e** > **3f**.

### 2.3. Reducing Power Ability

The synthesized compounds were further tested for their reducing power ability. Ferric ion reducing antioxidant power (FRAP) assay and cupric ion reducing antioxidant capacity (CUPRAC) were measured using butylated hydroxytoluene as the standard.

#### 2.3.1. Ferric Reducing Antioxidant Power (FRAP) Assay

**Figure 14:**
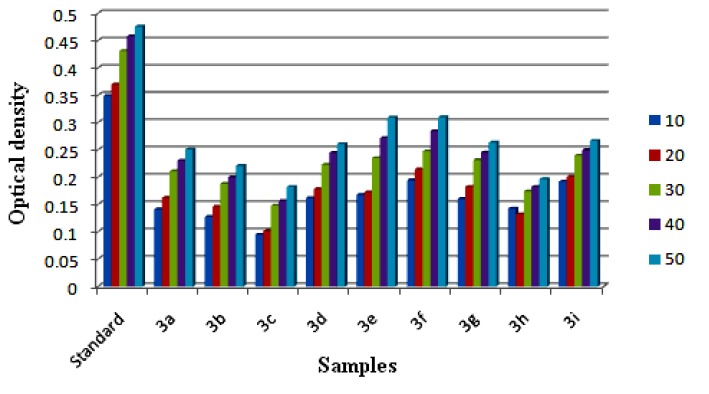
Ferric ion reducing power ability of samples **3**(**a**–**i**) at different concentration (10–50 μg/mL) measured at 700 nm. Values are expressed as absorbance; high absorbance indicates high reducing power.

In practice, the antioxidant activity of a substance is directly correlated to its reducing ability. Standard assays like FRAP provide a reliable method to verify the antioxidant ability of a substance. Substances having reduction potential react with potassium ferricyanide forming potassium ferrocyanide. The formed potassium ferrocyanide further reacts with FeCl_3_ to form an intense Prussian blue complex which has a maximum absorbance at 700 nm. The complex formed is directly proportional to the reducing capacity of the test sample. An increase in absorbance is equal to the reducing power of the sample. Results are depicted in Figure 14 and from the analysis it is clear that the compounds **3d**, **3e**, **3f** and **3i** showed good ferric reducing ability, whereas **3a**, **3g** and **3h** were moderate, and the compounds **3b** and **3c** showed the least reducing ability among the series.

#### 2.3.2. Cupric Ion Reducing Antioxidant Capacity (CUPRAC) Assay

**Figure 15:**
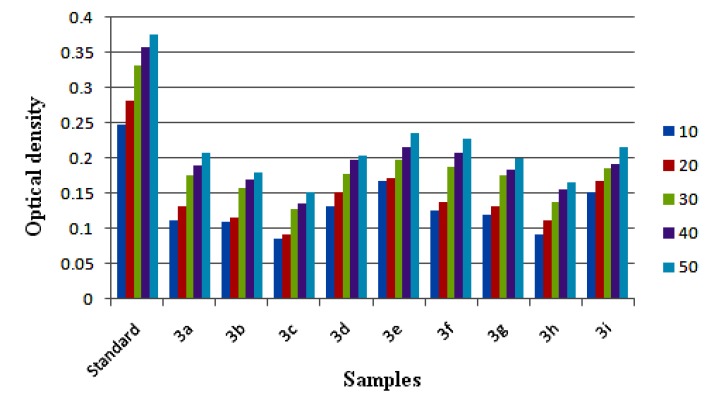
Cupric ion reducing power ability of samples **3**(**a**–**i**) at different concentration (10–50 μg/mL) measured at 700 nm. Values are expressed as absorbance; high absorbance indicates high reducing power.

In this assay a sample under evaluation effectively reduces Cu^2+^ to Cu^+^, changing the characteristic ion absorption. The reduced Cu^+^ ion combines with the chromogenic reagent neocuproine forming a stable 2:1 complex which has a maximum absorption at 450 nm. This method operates at pH 7. Results, shown in Figure 15, indicate that the majority of these test compounds have good reducing ability. These compounds displayed 40% less reducing power compared to the standard. Compounds **3d**, **3e**, **3f** and **3i** showed good cupric reducing ability, whereas compounds **3a**, **3g** and **3h** were moderate, and the compounds **3b** and **3c** showed the least reducing ability among the series.

## 3. Experimental

### 3.1. Materials and Method

Melting points were determined on a Stuart Scientific (UK) apparatus. The purity of each compound was confirmed by thin layer chromatography using Merck silica gel 60 F_254_-coated aluminium plates. The mass spectra were recorded on a Jeol JMS-D 300 mass spectrometer operating at 70 eV. Elemental analyses (CHN) were carried out on a Perkin Elmer Series II, 2400 analyzer. X-ray analysis was done using a Bruker SMART Apex II or Apex II Duo CCDC diffractometer. The data were processed with SAINT and absorption correction was done using SADABS [23]. The structures were solved by direct method using the program SHELXTL [24], and were refined by full-matrix least squares technique on *F^2^* using anisotropic displacement parameters. The non-hydrogen atoms were refined anisotropically. In these compounds, all the H atoms were calculated geometrically with isotropic displacement parameters set to 1.2 (1.5 for methyl groups) times the equivalent isotropic *U* values of the parent carbon atoms. The overlay structures were drawn using Olex^2^ software [25]. Crystallographic data for compounds **3**(**a**–**i**) have been deposited at the Cambridge Crystallographic Data Centre with CCDC No: 939875, 939876, 939877, 942741, 942742, 948856, 944072, 946445 and 948855, respectively. Copies of the data can be obtained free of charge on application to the CCDC, 12 Union Road, Cambridge CB2 IEZ, UK. Fax: +44-(0)1223-336033 or E-Mail: deposit@ccdc.cam.ac.uk.

### 3.2. General Procedure for the Synthesis of Chalcones **3**(**a**–**i**)

A mixture of 2-acetyl-5-chlorothiophene (0.01 mol) and a halogen-substituted benzaldehyde (0.01 mol) was dissolved in methanol (20 mL). Catalytic amount of NaOH was added to the solution drop-wise with vigorous stirring. The reaction mixture was stirred for about 5–6 h at room temperature. The resultant crude products were filtered, washed successively with distilled water and recrystallized from ethanol to get corresponding chalcones. Crystals suitable for X-ray diffraction studies were obtained by the slow evaporation technique using a suitable solvent.

*(2E)-1-(5-Chlorothiophen-2-yl)-3-(2-fluorophenyl)prop-2-en-1-one* (**3a**): Solvent for growing crystals: Mixture of acetone and ethanol (1:1 v/v); Yield: 64%; M.P. 112–114 °C; LCMS: *m/z =* 267 (M^+^+1); Elemental analysis: Calculated for C_13_H_8_ClFOS: C, 58.54%; H, 3.02%; Found: C, 58.51%; H, 3.11%. CCDC No.: 939875.

*(2E)-1-(5-Chlorothiophen-2-yl)-3-(3-fluorophenyl)prop-2-en-1-one* (**3b**): Solvent for growing crystals: Mixture of acetone and ethanol (1:1 v/v); Yield: 61%; M.P. 118–120 °C; LCMS: *m/z* = 267 (M^+^+1); Elemental analysis: Calculated for C_13_H_8_ClFOS: C, 58.54%; H, 3.02%; Found: C, 58.49%; H, 3.17%. CCDC No.: 939876.

*(2E)-1-(5-Chlorothiophen-2-yl)-3-(4-fluorophenyl)prop-2-en-1-one* (**3c**): Solvent for growing crystals: Mixture of acetone and ethanol (1:1 v/v); Yield: 68%; M.P. 128–130 °C; LCMS: *m/z* = 267 (M^+^+1); Elemental analysis: Calculated for C_13_H_8_ClFOS: C, 58.54%; H, 3.02%; Found: C, 58.51%; H, 3.13%. CCDC No.: 939877.

*(2E)-1-(5-Chlorothiophen-2-yl)-3-(3-chloro-4-fluorophenyl)prop-2-en-1-one* (**3d**): Solvent for growing crystals: Mixture of acetone, ethanol and acetonitrile (1:1:1 v/v); Yield: 63%; M.P. 167–169 °C; LCMS: *m/z* = 302 (M^+^+1); Elemental analysis: Calculated for C_13_H_7_Cl_2_FOS: C, 51.85%; H, 2.34%; Found: C, 51.79%; H, 2.48%. CCDC No.: 942741.

*(2E)-1-(5-Chlorothiophen-2-yl)-3-(3-bromo-4-fluorophenyl)prop-2-en-1-one* (**3e**): Solvent for growing crystals: Mixture of acetone, ethanol and acetonitrile (1:1:1 v/v); Yield: 60%; M.P. 144–146 °C; LCMS: *m/z* = 346 (M^+^+1); Elemental analysis: Calculated for C_12_H_7_BrClFOS: C, 45.18%; H, 2.04%; Found: C, 45.13%; H, 2.11%. CCDC No.: 942742.

*(2E)-1-(5-Chlorothiophen-2-yl)-3-(4-bromo-2-fluorophenyl)prop-2-en-1-one* (**3f**): Solvent for growing crystals: N,N-dimethylformamide; Yield: 62%; M.P. 133–134 °C; LCMS: *m/z* = 346 (M^+^+1); Elemental analysis: Calculated for C_12_H_7_BrClFOS: C, 45.18%; H, 2.04%; Found: C, 45.15%; H, 2.09%. CCDC No.: 948856.

*(2E)-3-(3-Chlorophenyl)-1-(5-chlorothiophen-2-yl)prop-2-en-1-one* (**3g**): Solvent for growing crystals: Mixture of acetone and ethanol (1:1 v/v); Yield: 70%; M.P. 120–122 °C; LCMS: *m/z* = 284 (M^+^+1); Elemental analysis: Calculated for C_13_H_8_Cl_2_OS: C, 55.14%; H, 2.85%; Found: C, 55.11%; H, 2.92%. CCDC No.: 944072.

*(2E)-1-(5-Chlorothiophen-2-yl)-3-(3,5-dichlorophenyl)prop-2-en-1-one* (**3h**): Solvent for growing crystals: Mixture of acetone, ethanol and ethyl acetate (1:1:1 v/v); Yield: 67%; M.P. 129–131 °C; LCMS: *m/z* = 318 (M^+^+1); Elemental analysis: Calculated for C_13_H_7_Cl_3_OS: C, 49.16%; H, 2.22%; Found: C, 49.13%; H, 2.29%. CCDC No.: 946445.

*(2E)-1-(5-Chlorothiophen-2-yl)-3-(2,6-dichlorophenyl)prop-2-en-1-one* (**3i**): Solvent for growing crystals: N,N-dimethylformamide; Yield: 64%; M.P. 170–172 °C; LCMS: *m/z* = 318 (M^+^+1); Elemental analysis: Calculated for C_13_H_7_Cl_3_OS: C, 49.16%; H, 2.22%; Found: C, 49.12%; H, 2.27%. CCDC No.: 948855.

### 3.3. *In Vitro* Antimicrobial Activities

#### 3.3.1. Antibacterial Activity

All the synthesized compounds were screened for their antibacterial activity against Gram-positive bacteria such as *Bacillus subtilis* MTCC 121 and *Staphylococcus aureus* MTCC 7443 and Gram-negative bacteria such as *Xanthomonas campestris* MTCC 7908 and *Escherichia coli* MTCC 7410 in DMF by disc diffusion method on nutrient agar medium [26]. Each Petri plate was filled with sterile medium (nutrient agar medium, 15 mL) uniformly and smeared with cultures of Gram-positive bacteria to which 50 µL (1 mg/mL: *i.e.*, 50 µg/disc) of the test compounds was added. The treatments also include 50 µL of DMF as negative control and bacteriomycin and gentamycin as positive control for comparisons. Three replicates were maintained for each treatment. The plates were incubated at 37 ± 2 °C for 24 h and the zone of inhibition was determined.

#### 3.3.2. Antifungal Activity

Antifungal activity of the synthesized compounds was screened against *Fusarium oxysporum* MTCC 2480 in DMF by the poisoned food technique [27]. Potato dextrose agar (PDA) medium was prepared; to each Petri plate PDA (15 mL) was added and allowed to solidify. A 5 mm disc of 7 day-old culture of the test fungi was placed at the center of the Petri plate and incubated at 26 °C for 7 days. After the incubation period, the percentage inhibition was measured and three replicates were maintained for each treatment. Nystatin was used as the standard. Synthesized compounds were tested at the dosage of 500 µL of the novel compounds/Petri plate, where the concentration was 0.1 mg/mL by poisoned food technique.

#### 3.3.3. Ferric Ion Reducing Antioxidant Power (FRAP) Assay

The synthesized compounds were screened for ferric reducing power antioxidant ability by the method reported by Oyaizu [28]. The method is based on the reduction of ferric (Fe^3+^) to ferrous (Fe^2+^), which is accomplished in presence of antioxidants. Samples of **3**(**a**–**i**) having a concentration of 10–50 μg/mL were mixed with an equal volume of 0.2 M phosphate buffer (pH 6.6) and 1% potassium ferricyanide and the mixture were incubated for 20 min at 50 °C. The mixture was acidified with 2.5 mL of 10% trichloroacetic acid and then centrifuged at 3,000 rpm for about 15 min. The upper supernatant liquid was diluted with distilled water and 0.1% ferric chloride was added. The absorbance was measured at 700 nm. The increase in absorbance is directly proportional to the reducing ability of the compound. The control was prepared as above without the sample.

#### 3.3.4. Cupric Ion Reducing Antioxidant Capacity (CUPRAC) Assay

The synthesized compounds were also evaluated for their cupric ion reducing power by a reported method [29]. CUPRAC is a widely applicable method for evaluating the antioxidant properties of a substance. A mixture of CuCl_2_ (1 mL, 0.01 M) solution, ethanolic neocuproine (1 mL, 0.0075 M) and ammonium acetate (1 mL, 1 M) were dissolved and 1 mL of test samples (10–50 μg/mL) was added along with 0.1 mL of distilled water. The mixture was incubated for about 30 min and the absorbance was measured at 450 nm against the blank solution. Control is prepared as above without the sample.

#### 3.3.5. Statistical Analysis

All the assay measurement were performed in triplicate (n = 3) and are expressed as mean of the three determinations.

## 4. Conclusions

Halogen-substituted chalcone derivatives **3**(**a**–**i**) bearing the 5-chlorothiophene moiety were synthesized in good yield and the structures of these compounds were determined by single crystal X-ray diffraction analysis. The crystal structure diversities of these compounds and various interactions responsible for their crystal stability are described. In addition, *in vitro* antimicrobial and reducing power ability of these compounds are evaluated. The reported compounds produced a varied range of inhibition results against the tested microbial strains, which is due to the presence of electron negative halogen(s) substituents at different positions on the phenyl ring. The antimicrobial activity of the tested compounds follows the order **3c** > **3a** > **3b** > **3g** > **3h** > **3i** > **3d** > **3e** > **3f**. These compounds displayed about 40% less reducing ability for ferric and cupric ions when compared to the standards. The present study demonstrated the relationship of the halogen linkage on the aromatic ring to the biological activities exhibited by these compounds.

## Supplementary Materials

Supplementary materials can be accessed at: http://www.mdpi.com/1420-3049/18/10/12707/s1.

## References

[1] Alcaraz L.E., Blanco S.E., Puig O.N., Tomas F., Ferretti F.H. (2000). Antibacterial activity of flavonoids against methicillin-resistant *Staphylococcus aureus* strains. J. Theor. Biol..

[2] Baviskar B.A., Baviskar B., Shiradkar M.R., Deokate U.A., Khadabadi S.S. (2009). Synthesis and antimicrobial activity of some novel benzimidazolyl chalcones. J. Chem..

[3] Echeverria C., Santibañez J.F., Donoso-Tauda O., Escobar C.A., Ramirez-Tagle R. (2009). Structural antitumoral activity relationships of synthetic chalcones. Int. J. Mol. Sci..

[4] Modzelewska A., Pettit C., Achanta G., Davidson N.E., Huang P., Khan S.R. (2006). Anticancer activities of novel chalcone and bis-chalcone derivatives. Bioorg. Med. Chem..

[5] Vogel S., Heilmann J. (2008). Synthesis, cytotoxicity, and antioxidative activity of minor prenylated chalcones from Humulus lupulus. J. Nat. Prod..

[6] Babasaheb P.B., Sachin A.P., Rajesh N.G. (2010). Synthesis and biological evaluation of nitrogen containing chalcones as possible anti-inflammatory and antioxidant agents. Bioorg. Med. Chem.Lett..

[7] Kim Y.H., Kim J., Park H., Kim H.P. (2007). Anti-inflammatory activity of the synthetic chalcone derivatives: Inhibition of inducible nitric oxide synthase-catalyzed nitric oxide production from lipopolysaccharide-treated RAW 264.7 cells. Biol. Pharm Bull..

[8] Ballesteros J.F., Sanz M.J., Ubeda A., Miranda M.A., Iborra S., Paya M., Alcaraz M.J. (1995). Synthesis and pharmacological evaluation of 2'-hydroxychalcones and flavones as inhibitors of inflammatory mediators generation. J. Med. Chem..

[9] Vogel S., Barbic M., Jürgenliemk G., Heilmann J. (2010). Synthesis, cytotoxicity, anti-oxidative and anti-inflammatory activity of chalcones and influence of A-ring modifications on the pharmacological effect. Eur. J. Med. Chem..

[10] Lopez S.N., Castelli M.V., Zacchino S.A., Domnguez J.N., Lobo G., Charris-Charris J., Cortes J.C.G., Ribas J.C., Devia C., Rodrguez A.M. (2001). *In vitro* antifungal evaluation and structure-activity relationships of a new series of chalcone derivatives and synthetic analogues, with inhibitory properties against polymers of the fungal cell wall. Bioorg. Med. Chem..

[11] Beom-Tae K., Kwang-Joong O., Jae-Chul C., Ki-Jun H. (2008). Synthesis of dihydroxylated chalcone derivatives with diverse substitution patterns and their radical scavenging ability toward DPPH free radicals. Bull. Korean Chem. Soc..

[12] Doan T.N., Tran T.-D. (2011). Synthesis, antioxidant and antimicrobial activities of a novel series of chalcones, pyrazolic chalcones, and allylic chalcones. Pharmacol. Pharm..

[13] Go M.L., Wu X., Liu X.L. (2005). Chalcones: An update on cytotoxic and chemoprotective properties. Curr. Med. Chem..

[14] Sivakumar P.M., Prabhakar P.K., Doble M. (2011). Synthesis, antioxidant evaluation, and quantitative structure-activity relationship studies of chalcones. Med. Chem. Res..

[15] Vogel S., Ohmayer S., Brunner G., Heilmann J. (2008). Natural and non-natural prenylated chalcones: Synthesis, cytotoxicity and anti-oxidative activity. Bioorg. Med. Chem..

[16] Lemar K.M., Turner M.P., Lloyd D. (2002). Garlic (Allium sativum) as an anti-Candida agent: A comparison of the efficacy of fresh garlic and freeze-dried extracts. J. Appl. Microbiol..

[17] Tomar V., Bhattacharjee G., Kamaluddina K. (2007). Synthesis and antimicrobial evaluation of newchalcones containing piperazine or 2,5-dichlorothiophene moiety. Bioorg. Med. Chem. Lett..

[18] Bag D., Ramar S., Degani M.S. (2009). Synthesis and biological evaluation of a, *b*-unsaturated ketone as potential antifungal agents. Med. Chem. Res..

[19] Tran T.D., Nguyen T.T., Do T.H., Huynh T.N., Tran C.D., Thai K.M. (2012). Synthesis and antibacterial activity of some heterocyclic chalcone analogues alone and in combination with antibiotics. Molecules.

[20] Ranganathan K., Arulkumaran R., Kamalakkannan D., Sundararajan R., Sakthinathan S.P., Vijayakumar S., Suresh R., Vanangamudi G., Thirumurthy K., Mayavel P. (2012). Silica-H_2_SO_4_ catalyzed environmentally benign crossed aldol condensation: Synthesis, spectral studies and biological activities of some 5-chloro-2-thienyl chalcones. Int. J. Pharm. Med. Biol. Sci..

[21] Kumar C.S.C., Loh W.-S., Ooi C.W., Quah C.K., Fun H.-K. (2013). Sructural correlation of some heterocyclic chalcone analogues and evaluation of their antioxidant potential. Molecules.

[22] Bernstein J., Davis R.E., Shimoni L., Chang N.L. (1995). Patterns in hydrogen bonding: Functionality and graph set analysis in crystals. Angew. Chem. Int. Edit. Engl..

[23] Bruker (2009). APEX2, SAINT and SADABS.

[24] Sheldrick G.M. (2008). A short history of SHELX. Acta Cryst..

[25] Dolomanov O.V., Bourhis L.J., Gildea R.J., Howard J.A.K., Puschmann H. (2009). OLEX2: A complete structure solution, refinement and analysis program. J. Appl. Cryst..

[26] Bauer W.M., Kirby J.C., Sherris, Truck M. (1966). Antibiotic susceptibility testing by a standardized single disk method. Am. J. Clin. Pathol..

[27] Satish S., Mohana D.C., Raghavendra M.P., Raveesha K.A. (2007). Antifungal activity of some plant extracts against important seed borne pathogens of *Aspergillus* sp.. J. Agric. Technol..

[28] Oyaizu M. (1986). Studies on products of browning reaction prepared from glucosamine. Jpn. J. Nutr..

[29] Apak R., Guclu K., Ozyurek M., Celik S.E. (2008). Mechanism of antioxidant capacity assays and the CUPRAC (cupric ion reducing antioxidant capacity) assay. Microchim. Acta.

